# Exploring TRIM proteins’ role in antiviral defense against influenza A virus and respiratory coronaviruses

**DOI:** 10.3389/fcimb.2024.1420854

**Published:** 2024-07-15

**Authors:** Ying Wei, Junzhu Song, Jingyu Zhang, Songbiao Chen, Zuhua Yu, Lei He, Jian Chen

**Affiliations:** ^1^ Laboratory of Functional Microbiology and Animal Health, College of Animal Science and Technology, Henan University of Science and Technology, Luoyang, China; ^2^ Luoyang Key Laboratory of Live Carrier Biomaterial and Animal Disease Prevention and Control, Henan University of Science and Technology, Luoyang, China; ^3^ The Key Lab of Animal Disease and Public Health, Henan University of Science and Technology, Luoyang, China

**Keywords:** TRIM proteins, influenza A virus, respiratory coronavirus, viral components, innate immunity

## Abstract

Numerous tripartite motif (TRIM) proteins, identified as E3 ubiquitin ligases, participate in various viral infections through ubiquitylation, ISGylation, and SUMOylation processes. Respiratory viruses, particularly influenza A virus (IAV) and respiratory coronaviruses (CoVs), have severely threatened public health with high morbidity and mortality, causing incalculable losses. Research on the regulation of TRIM proteins in respiratory virus infections is crucial for disease prevention and control. This review introduces TRIM proteins, summarizes recent discoveries regarding their roles and molecular mechanisms in IAV and CoVs infections, discusses current research gaps, and explores potential future trends in this rapidly developing field. It aims to enhance understanding of virus–host interactions and inform the development of new molecularly targeted therapies.

## 1 Introduction

Tripartite motif (TRIM) proteins are characterized by a conserved N-terminal RBCC motif, which comprises a RING zinc finger domain, one or two B-box domains, and a coiled-coil domain. Most TRIM proteins also possess a variable C-terminus and are found widely across insects, teleosts, and higher vertebrates (van der Aa et al., 2009; van Gent et al., 2018). The number of TRIM genes varies significantly across different species; for example, humans possess over 80 TRIM genes, chickens have approximately 54, zebrafish possess 208, and worms have 20 (Carthagena et al., 2009; Boudinot et al., 2011; Campbell et al., 2023). Our group has previously characterized the entire porcine TRIM family, identifying 57 porcine TRIM proteins (Wei et al., 2019). Notably, the key restriction factor TRIM22 was absent, suggesting that TRIM proteins are a rapidly evolving and species-specifically expanding polygenic family.

Despite their common domain features, TRIM proteins play multiple roles in diverse cellular processes, including development, differentiation, oncogenesis, apoptosis, and antiviral immunity (Ozato et al., 2008; Hatakeyama, 2017; Venuto and Merla, 2019). Growing interest in TRIM proteins research has highlighted these proteins as potent viral restriction factors. TRIM5, one of the best-studied anti-retroviral proteins, has been shown to inhibit post-entry stages of human immunodeficiency virus (HIV) and murine leukemia virus (MLV) (Stremlau et al., 2004; Pertel et al., 2011). Other TRIM proteins, such as TRIM11, TRIM25, and TRIM56, can also interfere with various stages of HIV-1 or MLV replication (Kane et al., 2016). TRIM22 has been reported to restrict numerous viruses by distinct mechanisms, including blocking the release of HIV Gag-only particles and the RNA synthesis of hepatitis B virus, inhibiting respiratory syncytial virus (RSV) replication by targeting JAK-STAT1/2 signaling, and interrupting herpes simplex virus 1 by epigenetic silencing of viral immediate-early genes (Barr et al., 2008; Gao et al., 2009; Reddi et al., 2021; Wang et al., 2021a). TRIM22 also suppresses the replication of severe acute respiratory syndrome coronavirus 2 (SARS-CoV-2) by ubiquitin-proteasome degrading NSP8 (Fan et al., 2024). It can interact with influenza A virus (IAV) and porcine reproductive and respiratory syndrome virus nucleoprotein for degradation (Di Pietro et al., 2013; Jing et al., 2019). Currently, more studies of TRIM proteins are focusing on the RING domain with E3 ubiquitin ligase activity.

Viral restriction by many TRIM proteins appears to be virus species- or family-specific, whereby multiple TRIM proteins contribute to the effective restriction of a particular virus. For example, porcine endogenous retroviruses are insensitive to divergent mammalian TRIM5α proteins, although they can strongly restrict various lentiviruses (Wood et al., 2009). More importantly, only a few TRIM proteins are known to restrict distinct viruses in a common manner. In addition, several TRIM proteins can also be utilized to benefit viral proliferation (Yang et al., 2022; Cui et al., 2023; Li X. et al., 2024). The global community is experiencing a severe public health crisis due to the co-circulation of respiratory viruses, notably IAV and SARS-CoV-2. Vaccines play a crucial role in combating infectious diseases, yet respiratory viruses pose unique challenges (Dadashi et al., 2021; Wang et al., 2022; Morens et al., 2023). Hence, it is of great significance to study the interaction between viruses and hosts and to identify novel antiviral factors, which will provide a basis for developing novel antiviral drug targets. Here, we will review the latest progress in the interplay between respiratory viruses and TRIM proteins, enriching the rationale of virus–host interaction and also providing unique insight into the role of the TRIM proteins in viral replication. Summary of the roles of TRIM proteins in influenza A virus and respiratory coronaviruses infections is shown in **Table 1**.

## 2 Influenza A virus

The influenza virus, an RNA virus from the Orthomyxoviridae family, poses significant health risks to humans and animals by causing mild to severe respiratory symptoms. Influenza viruses can be classified into four types, among which influenza A and B viruses are the most common pathogens in humans. The IAV is highly variable and pathogenic and can be further identified by different subtypes based on their surface proteins hemagglutinin (HA) and neuraminidase (NA), such as H1N1 and H3N2. Known for its wide range of animal host species, IAV infection has the potential to trigger global pandemics (Javanian et al., 2021; Berche, 2022). This highlights the critical importance of understanding its pathogenic characteristics and addressing the challenges in its control and prevention. The high frequency of mutation and gene reassortment in the IAV complicates efforts to combat it, as these genetic changes can lead to the emergence of new, more adaptable virus subtypes. Such variability underscores the necessity of identifying robust antivirals through the study of virus–host interactions. One promising area of research in this context is the role of TRIM proteins. These proteins are part of the host’s immune response and could potentially serve as new targets for antiviral strategies, offering new avenues for therapeutic intervention against the influenza virus.

### 2.1 TRIM-mediated IAV inhibition

#### 2.1.1 TRIM proteins directly antagonize viral components

Increasing data suggest that TRIM proteins can directly antagonize viral components to restrict IAV infection. One of the major targets of TRIM proteins is the nucleoprotein (NP), a major structural component of the viral ribonucleoprotein (vRNP). NP binding to viral RNA is crucial for vRNP activity during the viral transcription and replication processes (Turrell et al., 2013; Te Velthuis and Fodor, 2016). TRIM proteins, including TRIM14, TRIM22, and TRIM41, mediate the polyubiquitination and subsequent proteasomal degradation of NP (Di Pietro et al., 2013; Patil et al., 2018; Wu et al., 2019). TRIM14 could interact with the viral NP based on the PRY-SPRY domain and then facilitate the K48-linked ubiquitination of NP for proteasome-dependent degradation, leading to the inhibition of IAV RNP formation. TRIM14 could also effectively prevent the translocation of NP from the cytoplasm to the nucleus and further restrict IAV replication *in vitro* (Wu et al., 2019). TRIM22, as an IAV-induced gene and IFN-stimulated gene (ISG), strongly interrupted IAV replication in human alveolar epithelial A549 cells and MDCK cells. The TRIM22–NP interaction could induce the polyubiquitination-proteasome degradation of NP (Di Pietro et al., 2013). Interestingly, another study showed that TRIM22 could be constitutively expressed without viral infection or innate immune stimulation in primary cell lines and the airways of rhesus macaques. Constitutive TRIM22 expression was sufficient to inhibit viral transcription independently of IFN-mediated anti-IAV innate immune defense, which conferred a pre-existing defense against IAV infection. These research studies highlighted the intracellular restriction of IAV by TRIM22 in what seemed to be tissue- or cell-specific patterns (Charman et al., 2021). The proteomic study revealed that TRIM41 interacted with NP through its SPRY domain and targeted NP for proteasomal destruction dependent on its E3 ligase activity. Ectopic TRIM41 expression decreased host susceptibility to IAV infection. Conversely, RNA interference (RNAi) and knockout of TRIM41 facilitated IAV infection (Patil et al., 2018).

Apart from its intrinsic defense role, TRIM21 could inhibit the replication of H3/H5/H9 IAV subtypes by targeting matrix protein 1 (M1), while being ineffective against H1 and H7 M1. TRIM21 bound to the residue R95 of M1 and promoted the K48-linked ubiquitination and proteasomal degradation of M1 at K242. The M1 R95K or K242R mutations conferred resistance to TRIM21, leading to more effective replication and severe pathogenicity. Interestingly, a TRIM21-driven host adaptive R95K mutation was found when avian influenza virus spilled over to mammals (Lin et al., 2023). Affinity purification coupled with mass spectrometry was used to identify host factors interacting with polymerase basic protein 1 (PB1), the catalytic core of the IAV RNA polymerase complex. Data showed that TRIM32 could interact with multiple IAV strains’ PB1 and promote the translocation of PB1 to the nucleus. Subsequently, TRIM32 inhibited the polymerase activity by ubiquitinating the PB1. Reconstitution of trim32^−/−^ mouse embryonic fibroblasts with TRIM32, but not a catalytically inactive mutant, restored viral restriction (Fu et al., 2015).

TRIM25 has been demonstrated to be an RNA-binding protein, which plays a key role in the antiviral interferon response by activating the RIG-I pathway (Choudhury et al., 2020; Xiao et al., 2021; Diaz-Beneitez et al., 2022). However, new evidence has shown alternative mechanisms for TRIM25 anti-IAV action. For example, activating the RIG-I pathway did not require TRIM25 activity in human-derived cultured cells upon IAV infection. TRIM25 bound and destabilized IAV mRNAs to downregulate the targeted RNA rather than directly inhibiting IAV transcription. Surprisingly, the previously identified RNA-binding domain and the E3 ubiquitin ligase domain were redundant for inhibiting IAV replication (Choudhury et al., 2022). Another study indicated a nuclear role for TRIM25 in suppressing IAV replication independent of ubiquitin ligase activity and IFN response. Nuclear TRIM25 specifically targeted vRNP to inhibit RNA synthesis. Notably, instead of interrupting the initiation of the capped-RNA-primed viral mRNA synthesis, TRIM25 blocked the onset of RNA chain elongation because it could limit RNA transferring into the polymerase complex (Meyerson et al., 2017). Influenza A and B viruses were inhibited by TRIM56 in cell culture, while human metapneumovirus was not inhibited. Unlike its antiviral action against positive-strand RNA viruses, TRIM56 inhibition of influenza virus was independent of N-terminal domains (Liu et al., 2014). Rather, the expression of a 63-residue-long C-terminal tail portion of TRIM56 inhibited the replication of influenza viruses as effectively as that of full-length TRIM56 by impeding viral RNA synthesis. These data revealed that TRIM56 has developed multiple domains to inhibit distinct viral infections (Liu et al., 2016). Taken together, it is summarized that diverse TRIM proteins play a crucial role as intrinsic antiviral factors, either by directly impeding activities of influenza viral proteins, such as NP, M1, and PB1, or by impeding viral RNA synthesis.

#### 2.1.2 TRIM proteins positively regulate antiviral innate immunity

The innate immune system is the first line of defense against various pathogens. The conserved pathogen-associated molecular patterns (PAMPs) are recognized by a series of germline-encoded pattern recognition receptors (PRRs), including Toll-like receptors (TLRs), NOD-like receptors (NLRs), RIG-I-like receptors (RLRs), and cytosolic DNA-sensing receptors. Among them, the RLRs (particularly the RIG-I and MDA5) function as essential sensors of viral double-stranded RNA (dsRNA) or single-stranded RNA (ssRNA) in the cytoplasm (van Gent et al., 2018). Upon recognition, they recruit downstream signaling molecules for phosphorylation, which ultimately leads to the activation of cytoplasmic transcription factors such as nuclear NF-κB and interferon regulatory factors (IRFs) into the nucleus. This event promotes the transcription of diverse downstream immune-regulatory genes, including pro-inflammatory cytokines and chemokines. Importantly, IFN-I further activates the downstream JAK-STAT signaling pathway to induce the transcription of hundreds of ISGs (Bowie and Unterholzner, 2008; Wei et al., 2018; Shen et al., 2021). Not only can TRIM proteins be induced by IFN-I, but they can also modulate IFN-I, playing a significant regulatory role in antiviral infection and innate immune signaling pathways.

Well-characterized TRIM proteins in positively regulating anti-IAV innate immunity include TRIM21, which was upregulated in clinical patient serum and A549 cells upon IAV infection. Overexpression of TRIM21 reduced IAV replication correlated with increased IFN-α and IFN-β expression. Additionally, overexpression of TRIM21 decreased H1N1-induced inflammation and apoptosis by activating the TBK1-IRF3 signaling pathway in A549 cells (Yuan et al., 2024). In addition, a novel mechanism of PKR-mediated IFN-β expression was revealed in TRIM28 anti-HPAIV action. TRIM28 corepressor activity was specifically regulated by phosphorylation of S473, which resulted in increased expression of IFN-β, IL-6, and IL-8 during infection with HPAIV. Strain-specific phosphorylation of TRIM28 S473 was induced by a PKR signaling cascade in response to the viral RNA sensor RIG-I. Thus, TRIM28, as a critical positive immune regulator, protected against IAV infection by activating PKR-mediated IFN-β expression (Krischuns et al., 2018). *In vitro* and *in vivo* findings suggested novel roles of TRIM35 defense against IAV infection through activating TRAF3 and degrading viral PB2. TRIM35 mediated TRAF3 activation by catalyzing Lys63-linked ubiquitination, leading to the promotion of a signaling complex with VISA and TBK1 formation. IAV PB2 polymerase countered the RIG-I-mediated antiviral immunity by impeding TRAF3 activation. However, TRIM35 could degrade IAV PB2 via catalyzing Lys48-linked ubiquitination, thereby antagonizing its suppression of TRAF3 activation (Sun et al., 2020).

### 2.2 TRIM-mediated IAV promotion

As noted above, the TRIM proteins can also act as negative regulators of the PRR-mediated innate immune signaling pathway. For example, TRIM46 was significantly increased in response to H7N9 infection in A549 cells and accelerated IAV infection. TRIM46 mediated H7N9 inhibition with increased levels of phosphorylated IRF3 and IFN-I production in TRIM46-silenced cells. Conversely, TRIM46 mediated H7N9 promotion with decreased IFN-I production in TRIM46-overexpressed cells. TRIM46 was identified as a key negative regulator in the RIG-I-mediated antiviral immunity by catalyzing K48-linked ubiquitination of TBK1 (Su et al., 2022). Systems genetics of IAV-infected mice identified TRIM21 as a critical regulator of pulmonary innate immune response. The lung transcriptome data revealed a TRIM21-associated gene regulatory network response to IAV infection. TRIM21 transcripts were significantly upregulated in infected mice, whose expression may be regulated by Nr1d2 and Il3ra. Pathway analysis found TRIM21 involved in inflammation- and immunity-related signaling pathways. Subsequently, knockdown of TRIM21 in A549 cells led to significantly augmented levels of IAV-induced expression of IFNB1, IFNL1, CCL5, CXCL10, and ISGs (Li ZA. et al., 2024). Another identified negative regulator in the control of innate immune response in alveolar macrophages was TRIM29. The challenge of Trim29^−/−^ mice with influenza virus showed an improved survival rate due to increased production of IFN-I by macrophages. Mechanistically, TRIM29 directly targeted adaptor NEMO for ubiquitination and degradation, subsequently resulting in inhibiting interferon signaling via the transcription factor NF-κB (Xing et al., 2016).

## 3 Coronavirus

Since the 1990s, the cross-species transmission of multiple coronaviruses has severely threatened public health and caused acute respiratory syndrome, and even death in humans (Santos-López et al., 2021; Kesheh et al., 2022; Tang et al., 2022). In particular, the pandemic of coronavirus disease 2019 (COVID-19), caused by SARS-CoV-2, has further complicated the landscape, with at least 770 million cases and approximately 7 million deaths reported globally to date (W.H.O, 2024). Coronavirus (CoV) is an enveloped, single positive-stranded RNA virus belonging to the order Nidovirales, family Coronaviridae. Based on genetic characteristics and serological differences, coronaviruses may be divided into four categories: α-CoVs, β-CoVs, γ-CoVs, and δ-CoVs (Flores-Vega et al., 2022). Options to treat coronavirus are limited, and drug-resistant coronavirus strains can emerge through minor genetic changes. Meanwhile, the virus–host interaction is a dynamic and evolving process that influences the pathogenicity and host specificity of the virus. Thus, there is an urgent need to find novel anti-coronavirus effector proteins and develop therapeutic drugs based on these mechanisms.

### 3.1 TRIM-mediated coronavirus inhibition

#### 3.1.1 TRIM proteins directly antagonize viral components

Immunoprecipitation coupled with mass spectrometry was used to identify host factors interacting with SARS-CoV-2 nucleocapsid (N) protein. Data showed that the E3 ubiquitin ligase TRIM21 could interact with SARS-CoV-2 NP and polyubiquitinate it at Lys375. Subsequent degradation of NP led to the failure of SARS-CoV-2 viral particle assembly. This phenomenon has also been observed in SARS-CoV-2 variants, including α, β, γ, δ, and Omicron, together with severe acute respiratory syndrome coronavirus (SARS-CoV) and Middle East respiratory syndrome coronavirus (MERS-CoV) variants (Mao et al., 2023). Non-structural proteins play a critical role in SARS-CoV-2 replication, of which NSP7 and NSP8 served as subunits promoting the activity of RNA-dependent RNA polymerase (RdRp) of NSP12. All subunits comprising the RdRp complex were conducted to investigate the stability. The results showed that NSP8 was a relatively unstable protein, which could be readily recognized by the E3 ubiquitin ligase TRIM22. TRIM22 was induced upon SARS-CoV-2 infection and mediated Lys48-linked ubiquitination and proteasomal degradation of NSP8 at Lys97 (Fan et al., 2024). TRIM56 has been demonstrated to inhibit various virus replications, such as influenza virus, human coronavirus (HCoV) OC43, yellow fever virus (YFV), and dengue virus serotype 2 (DENV2) (Liu et al., 2014; Liu et al., 2016). TRIM56’s anti-IAV effect only depended on the expression of a short C-terminal tail portion. Rather, TRIM56 inhibition of positive-strand RNA viruses was always dependent on the N-terminal RING domain. Notably, TRIM56-mediated inhibition of HCoV-OC43 relied solely on its E3 ligase activity, whereas its restriction of YFV/DENV2 required both the E3 ligase activity and the integrity of the C-terminal portion. Furthermore, TRIM56 impeded intracellular IAV/YFV/DENV2 RNA synthesis, while it interfered at a later step in the HCoV-OC43 life cycle, suggesting the antiviral broad spectrum and diversity of TRIM56.

#### 3.1.2 TRIM proteins modulate ACE2 expression

SARS-CoV-2 binds to angiotensin-converting enzyme 2 (ACE2) to enter into host cells. TRIM28 was validated as a novel regulator of ACE2 expression and SARS-CoV-2 cell entry. Knockdown of TRIM28 upregulated IFN-γ receptor 2 expression and enhanced IFN-γ-induced ACE2 expression in both A549 and primary pulmonary alveolar epithelial cells. However, the upregulated ACE2 was partially reversed by dexamethasone in A549 cells, which may aid in developing a specific COVID-19 treatment target (Wang et al., 2021b).

### 3.2 TRIM-mediated coronavirus promotion

The host-mediated post-translational modifications of viral proteins have been shown to be an important strategy for regulating virus proliferation. TRIM6 facilitated SARS-CoV-2 proliferation by catalyzing the K29-linked ubiquitination of NP to enhance the ability to bind viral genomes (Zhou et al., 2024). TRIM28-mediated NP SUMOylation enhanced SARS-CoV-2 virulence (Ren et al., 2024). Mechanically, TRIM6 could interact with NP’s CTD via its RBCC domains and K29-linked ubiquitinate NP at K102, K347, and K361, increasing its binding to viral genomic RNA. Notably, the relatively conserved NP of SARS-CoV can also be ubiquitinated by TRIM6, indicating that NP could be a broad-spectrum anti-coronavirus target. TRIM28 SUMOylated SARS-CoV-2 NP at Lys65. Subsequent mediation of homo-oligomerization, RNA association, and liquid–liquid phase separation led to robust immunosuppression. This phenomenon has been further increased in SARS-CoV-2 NP R203K mutation with a novel site of SUMOylation. Surprisingly, an interfering peptide blocked the TRIM28-NP interaction by impeding NP SUMOylation, ultimately rescuing antiviral immunity.

## 4 Discussion

It has become clear that many TRIM proteins serve as commanders in various virus infections. As reviewed here, we have highlighted their critical roles in combating respiratory virus replication either by directly targeting specific viral components or by modulating host immune responses (e.g., interferon-dependent antiviral pathways or autophagy). Elucidating the interplay between TRIM proteins and viruses has promisingly opened new opportunities for respiratory virus therapeutic development. However, a significant challenge in clinical applications of TRIM proteins is their multifunctional properties. First, TRIM-mediated inhibition seems to be virus family- or even strain-specific. Human metapneumovirus was insensitive to TRIM56 even though it could strongly restrict replication of various viruses, such as the influenza virus and the human coronavirus (Liu et al., 2014; Liu et al., 2016). TRIM21 inhibited replication of H3/H5/H9 IAV subtypes by ubiquitination-dependent degradation of M1, while being ineffective against H1 and H7 M1 (Lin et al., 2023). Second, several TRIM proteins have more than one antiviral action. One prominent example is the RNA-binding protein TRIM25, whose role in the activation of the RIG-I is well demonstrated. Recent evidence has shown alternative mechanisms for TRIM25’s anti-IAV function independent of ubiquitin ligase activity and IFN response. On the one hand, TRIM25 could destabilize IAV mRNAs to restrict IAV replication (Choudhury et al., 2022). On the other hand, nuclear TRIM25 specifically targeted IAV ribonucleoproteins by blocking the onset of RNA chain elongation to inhibit RNA synthesis (Meyerson et al., 2017). Third, only a few TRIM proteins restrict distinct viruses in a common way. TRIM56 has developed multiple domains to inhibit IAV, HCoV-OC43, YFV, and DENV2 infections. Meanwhile, TRIM56 impeded intracellular IAV/YFV/DENV2 RNA synthesis, while interfering at a later step in the HCoV-OC43 life cycle (Liu et al., 2014; Liu et al., 2016). Another well-characterized antiviral TRIM protein is the E3 ubiquitin ligase TRIM22. TRIM22 restricted IAV and SARS-CoV-2 replication through ubiquitin-proteasome degrading NP and NSP8, respectively (Di Pietro et al., 2013; Fan et al., 2024). Rather, TRIM22 suppressed RSV replication by targeting JAK-STAT1/2 signaling (Wang et al., 2021a). Fourth, TRIM proteins have dual roles in regulating virus infection. In the case of SARS-CoV-2, knockdown of TRIM28 could facilitate SARS-CoV-2 cell entry by regulating ACE2 expression (Wang et al., 2021b). In contrast, TRIM28-mediated NP SUMOylation enhanced SARS-CoV-2 virulence (Ren et al., 2024). Hence, going forward in understanding the altered expression of TRIM proteins and their potential antiviral mechanisms, we should address some significant gaps in knowledge regarding the crystal structure and post-translational modifications’ regulation of TRIM proteins.

Recent studies have identified certain viral proteins as antagonists of TRIM proteins to block their antiviral properties. The mechanisms by which viruses circumvent TRIM-mediated antiviral functions to facilitate infection are still elusive. TRIM25-mediated RIG-I activation, whose mechanisms were thought to be well understood, has been demonstrated to be suppressed by viral proteins, including IAV NS1/PB1, SARS-CoV-2 NP/ORF6/NSP8, and MERS-CoV NP (Gack et al., 2009; Rajsbaum et al., 2012; Hu et al., 2017; Ban et al., 2018; Koliopoulos et al., 2018; Chang et al., 2020; Cheung et al., 2020; Jureka et al., 2020; Gori Savellini et al., 2021; Oh and Shin, 2021; Evseev et al., 2022; Tanaka et al., 2022; Khatun et al., 2023; Zhang et al., 2023).

In conclusion, TRIM proteins are rapidly evolving multifunctional proteins experiencing species-specific expansion. Some TRIM proteins are beginning to employ common antiviral strategies. Future research on the diverse aspects of virus–TRIM interactions will be crucial in determining the relevance of antiviral TRIM proteins across different tissues and host species. Further investigation into the mechanisms of TRIM proteins holds promise for developing them into inhibitors of viral replication, underscoring the potential of targeting host factors associated with viral replication as a suitable antiviral strategy.

**Table 1:** Summary of the roles of TRIM proteins in influenza A virus and respiratory coronaviruses infections.

TRIM proteins	Structure	Virus	Antiviral function	Molecular mechanism	Ref.
TRIM6	R-B2-CC-PRY-SPRY	SARS-CoV-2	Promotion	Catalyze NP ubiquitination	(Zhou et al., 2024)
TRIM14	B2-CC-PRY-SPRY	IAV	Inhibition	Target NP for degradation	(Wu et al., 2019)
TRIM21	R-B2-CC-PRY-SPRY	IAV	Inhibition	Target M1 for degradation	(Lin et al., 2023)
		IAV	Inhibition	Target TBK1-IRF3 signaling	(Yuan et al., 2024)
		IAV	Promotion	Regulate innate immunity	(Li ZA. et al., 2024)
		SARS-CoV-2	Inhibition	Target NP for degradation	(Mao et al., 2023)
TRIM22	R-B2-CC-SPRY	IAV	Inhibition	Target NP for degradation	(Di Pietro et al., 2013)
		IAV	Inhibition	A pre-existing defense	(Charman et al., 2021)
		SARS-CoV-2	Inhibition	Target NSP8 for degradation	(Fan et al., 2024)
TRIM25	R-B1-B2-CC-PRY-SPRY	IAV	Inhibition	Destabilize viral mRNA	(Choudhury et al., 2022)
		IAV	Inhibition	Target viral ribonucleoproteins	(Meyerson et al., 2017)
TRIM28	R-B1-B2-CC-PHD-BROMO	IAV	Inhibition	Activate PKR-mediated IFN-β expression	(Krischuns et al., 2018)
		SARS-CoV-2	Inhibition	Target ACE2	(Wang et al., 2021b)
		SARS-CoV-2	Promotion	Catalyze NP SUMOylation	(Ren et al., 2024)
TRIM29	B2-CC	IAV	Promotion	Target NEMO for degradation	(Xing et al., 2016)
TRIM32	R-B2-CC-NHL	IAV	Inhibition	Target PB1 for degradation	(Fu et al., 2015)
TRIM35	R-B2-CC-PRY-SPRY	IAV	Inhibition	Activate TRAF3 and degrade PB2	(Sun et al., 2020)
TRIM41	R-B2-CC-PRY-SPRY	IAV	Inhibition	Target NP for degradation	(Patil et al., 2018)
TRIM46	R-B2-CC-Cos-FN3-SPRY	IAV	Promotion	Target TBK1 for degradation	(Su et al., 2022)
TRIM56	R-B1-B2-CC	IAV/YFV/DENV2	Inhibition	Restrict viral RNA synthesis	(Liu et al., 2014; Liu et al., 2016)
		HCoV-OC43	Inhibition	Disturb a later life cycle	(Liu et al., 2014)

* R, ring finger domain; B1, B-box1 domain; B2, B-box2 domain; CC, coiled-coil domain; PRY, ryanodine receptor; SPRY, SplA and ryanodine receptor; PHD, plant homeodomain; BROMO, bromodomain; NHL, NHL repeats; FN3, fibronectin type III motif.

## Author contributions

YW: Conceptualization, Funding acquisition, Writing – original draft, Writing – review & editing. JS: Writing – review & editing, Writing – original draft. JZ: Writing – review & editing, Writing – original draft. SC: Writing – review & editing. ZY: Writing – review & editing. LH: Writing – review & editing. JC: Writing – review & editing.
